# Anesthetic protocols for urodynamic studies of the lower urinary tract in small rodents—A systematic review

**DOI:** 10.1371/journal.pone.0253192

**Published:** 2021-06-24

**Authors:** Abdelkhalek Samy Abdelkhalek, Haroun Ali Youssef, Ahmed Sayed Saleh, Peter Bollen, Peter Zvara

**Affiliations:** 1 Biomedical Laboratory, Department of Clinical Research, Faculty of Health Sciences, University of Southern Denmark, Odense, Denmark; 2 Department of Surgery, Anesthesiology & Radiology, Veterinary Teaching Hospital, Faculty of Veterinary Medicine, Assiut University, Assiut, Egypt; 3 Research Unit of Urology, Department of Urology, Odense University Hospital, Odense, Denmark; University Medical Center Utrecht, NETHERLANDS

## Abstract

Urodynamic studies in rats and mice are broadly used to examine pathomechnisms of disease and identify and test therapeutic targets. This review aims to highlight the effects of the anesthetics on the lower urinary tract function and seeks to identify protocols that allow recovery from anesthesia and repeated measurements while preserving the function which is being studied. All studies published in English language, which compared the data obtained under various types of anesthesia and the urodynamics performed in awake animals were included. It appears that urethane, an anesthetic recommended extensively for the investigation of lower urinary tract function, is appropriate for acute urodynamic studies only. Major advantages of urethane are its stability and ability to preserve the micturition reflex. Due to its toxicity and carcinogenicity, urethane anesthesia should not be used for recovery procedures. This review evaluated available alternatives including propofol, isoflurane and combinations of urethane, ketamine/xylazine, ketamine/medetomidine, and/or fentanyl/fluanisone/midazolam. Different effects have been demonstrated among these drugs on the urinary bladder, the urethral sphincter, as well as on their neuroregulation. The lowest incidence of adverse effects was observed with the use of a combination of ketamine and xylazine. Although the variations in the reviewed study protocols represent a limitation, we believe that this summary will help in standardizing and optimizing future experiments.

## Introduction

Small rodent urodynamic studies contribute to our understanding of the pathophysiological processes of the lower urinary tract (LUT) and help identify targets for therapy of diseases such as overactive bladder, detrusor underactivity and urinary incontinence [1]. Small rodents, such as mice and rats, have many anatomical and physiological similarities to humans, their normal urinary function has been studied extensively in vitro and in vivo, and their use is cost effective. Their short lifespan facilitates investigation of the effects of aging on the LUT [2]. There are some significant differences between rat, mouse and human LUT function that must be considered when interpreting the data.

Urodynamics performed under conscious, free-moving conditions enable the collection of measurements without the effects of anesthesia. These measurements, however, cause stress to the animals and are often associated with movement artifacts. Motion artifact can be reduced by restraint, which in turn was shown to affect the voiding parameters and cause additional stress to the animal [1, 3, 4]. Anesthesia facilitates stable pressure recording, without movement artifacts, and allows for the collection of some measurements, such as leak point pressure (LPP), which are impossible in a non-sedated animal. Studies have indicated that anesthesia can affect the urodynamic parameters through selective effects on nervous system, smooth and striated muscle. Though these effects cannot be avoided, understanding of the mechanism of action of different anesthetic protocols will help limit them. This review summarizes existing literature addressing the effects of anesthesia on the LUT.

## Materials and methods

### I. Research strategy

A systematic review of English language literature published on PubMed, Science Direct, CrossRef Metadata, Google Scholar, and Google was carried out, using the following keywords: Urodynamics, cystometry, anesthesia, analgesia, lower urinary tract, rodents, mice and rats. The databases were searched and the relevant literature describing experiments which compared the effects of anesthetics on the lower urinary tract (LUT) function in rats and mice was summarized.

The systematic review focuses on the effects of the anesthetics previously used during evaluation of the LUT function. We reviewed publications which met the following criteria:

### II. Inclusion criteria

1. The original articles on rodent urodynamic studies published between 1951 to 2020 in English language.
2. Data on functional evaluation of voiding efficiency, bladder pressure during filling and micturition, external urethral sphincter myographic activity and function, and LPP measurements.
3. Adequate information on the animal species, sex, dose and route of anesthetic administration.
4. Description of selective effects of the anesthetic on individual urodynamic parameters.
5. Comparison between the LUT function under different types of anesthesia and/or to data obtained from control unanesthetized animals.

### III. Exclusion criteria

1. Anesthetics used merely for inducing anesthesia for the surgical procedures.
2. Studies that used other drugs that might have interacted with the effects of the anesthetic.

Studies which assessed the effects of anesthesia on LUT by comparing the data obtained under anesthesia to a control group of urodynamic recordings performed in non-anesthetized animals and studies which compared the LUT function recorded under two or more types of anesthesia, were included in the final analysis. Following parameters were compared: Filling pressure, threshold pressure, micturition pressure, functional bladder capacity, LPP, urethral sphincter bursting activity and sphincter electromyography. In addition, the systemic effects on cardiovascular and respiratory systems and urine output were summarized from the studies which reported them. Publication bias of this review was controlled for by including all studies which fulfilled the inclusion/exclusion criteria. The possible reporting bias of the individual studies has not been evaluated due to significant variations in the research protocols. All studies containing relevant data were included and the differential effects of various anesthetics and their combinations on bladder capacity, filling and voiding pressure, LPP and EUS EMG activity were summarized.

## Results

**Fig 1:**
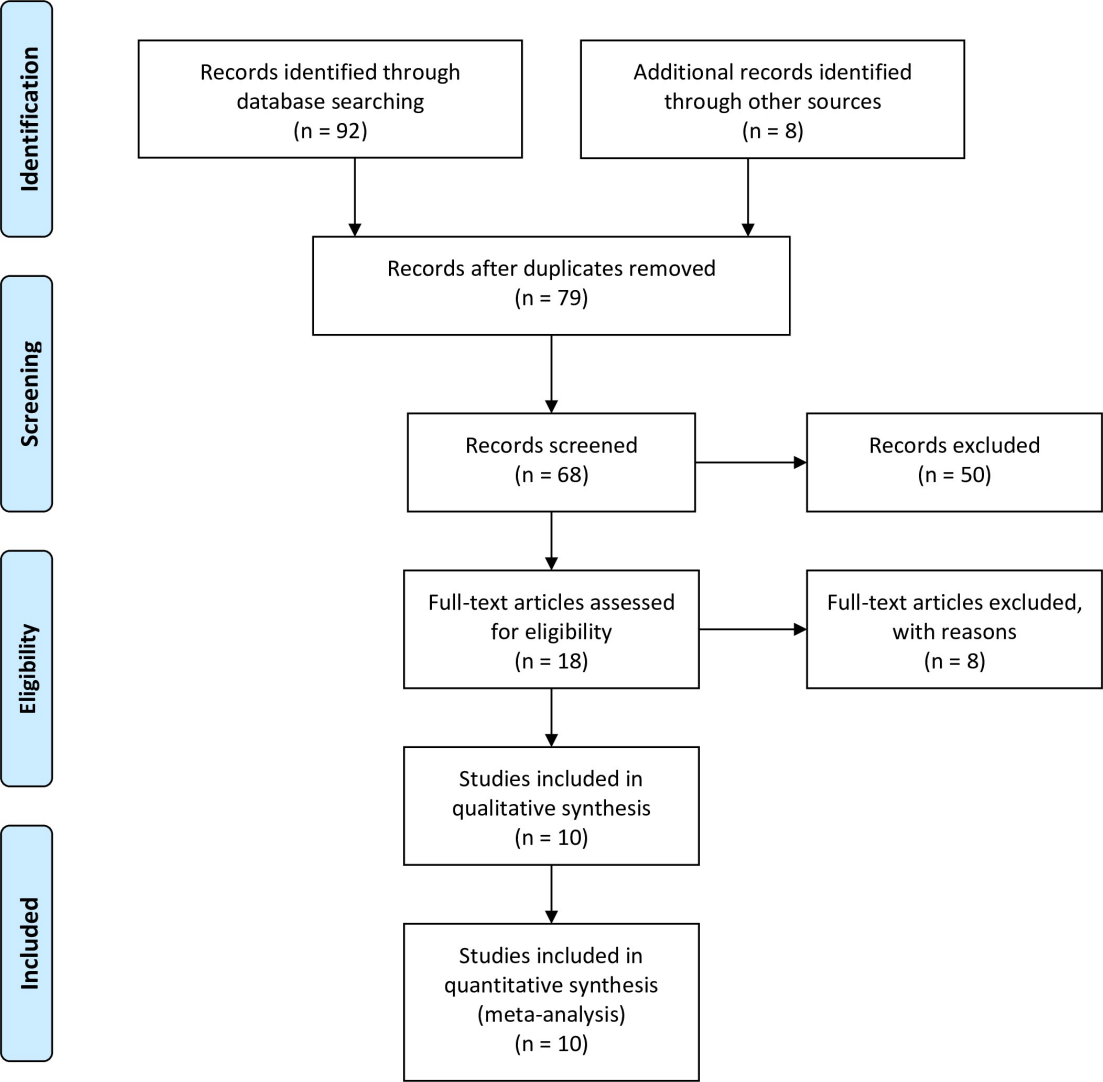
Flow diagram of data sources, screening, and inclusion.

The total number of studies identified in the database was 92. In addition, relevant information was identified in 8 book chapters. The total number of studies which reported data from urodynamics was 68. Fifty articles did not include comparison between the awake and anesthetized urodynamics or between different types of anesthesia and were therefore excluded from the final analysis. Additional 8 articles were excluded for following reasons; they used the anesthesia only during the surgical procedures or they used additional pharmacological interventions which could have interfered with the effects of the general anesthetic (Fig 1). Ten studies were included in the final analysis. General and spinal anesthetic protocols were reviewed separately.

### I. General anesthesia

Several anesthesia drugs or drug combinations can be applied using injection or inhalation [5, 6]. The choice of a specific anesthetic agent or anesthetic technique depends on factors such as potential interactions with research protocols or the necessary depth of anesthesia [7, 8].

#### 1. Urethane

Urethane is the ethyl ester of carbamic acid. It is readily soluble in water, alcohol and lipids. It potentiates the functions of neuronal nicotinic acetylcholine, gamma-aminobutyric acid (GABA)-A, a ligand-gated ion channel and a major inhibitory neurotransmitter in the central nervous system (CNS), and glycine receptors. In addition, it inhibits N-methyl-D-aspartate (NMDA) and α-amino-3-hydroxy-5-methyl-4-isoxazole propionic acid receptors. In concentrations inducing surgical anesthesia, urethane exerts modest effects on all ion channels and markedly depresses the dorsal root-evoked ventral root potentials [9–11]. Urethane produces little or no enhancement [9, 12], or inhibition [13] of GABAergic neurotransmission in the central and peripheral nervous systems. The frequent and continued use of urethane in neurophysiologic studies is due to its relatively minor effects on neurotransmission [14], cardiovascular function and its ability to produce relatively long, stable anesthesia following a single dose [11, 15].

In rats and mice, urethane anesthesia is commonly used in studies of the respiratory function, motility of the intestinal tract and on LUT function. Urethane produces a variety of side-effects at the endocrine and renal level [9] and has immunosuppressive properties [16].

In the research of LUT function, urethane was used in both rats and mice because it was believed to influence the voiding function less than other agents [17]. Smith and Kuchel compared LUT function in awake and urethane-anesthetized mice. The dose used was 1.2 g/kg subcutaneously (s.c.). The effect of urethane on mouse voiding was limited to delayed voiding pressure threshold and decreased micturition volume. Once activated, the amplitude of the voiding contraction was unchanged and micturition reflexes were intact and comparable between the two experimental groups [18]. Van Asselt and colleagues recommended urethane as an anesthetic for electrophysiological experiments on the LUT in rats when conscious animals cannot be used [19]. They advised starting with 1 g/kg (∼2/3 intraperitoneally (i.p.) and ∼1/3 s.c.) and administering additional urethane in small doses when necessary. In contrast, the studies using rats 6–8 weeks after spinal cord transection indicated that urethane anesthesia can suppress the micturition reflex, non-voiding bladder contractions during filling [20], and external urethral sphincter (EUS) electromyography (EMG) bursting activity during voiding [21]. This observation was made with the use of two different doses: 1.2 or 0.8 g/kg s.c. Another study by Yoshiyama et al. [22] employed a decerebrated rat model and suggested that urethral activity is more sensitive to the suppressive effect of urethane than afferent or efferent mechanisms controlling the bladder. Furthermore, the afferent limb of the micturition reflex pathway had a higher sensitivity to urethane than the efferent limb. The effects of urethane persisted after removal of the forebrain suggesting actions on the brain stem, spinal cord and peripheral nervous system.

#### 2. Fentanyl, fluanisone and midazolam

The combination of fentanyl, fluanisone (Hypnorm^®^) and midazolam, often referred to as neuroleptanalgesic combination, consists of the potent opioid analgesic, fentanyl, combined with the butyrophenone fluanisone, and benzodiazepine midazolam. Opioids provide excellent analgesic effects, but result in opioid rigidity, which has to be counteracted by fluanisone, a tranquilizer preventing excitation of the CNS [19] and midazolam, a muscle relaxant [23].

Fentanyl is a synthetic phenylpiperidine derivative. It is 100 times more potent than morphine and predominantly metabolized in the liver [24]. Fluanisone was previously used as an antipsychotic drug [25, 26]. When combined with diazepam, it causes profound hypotension in rats [27]. Midazolam is a short-acting benzodiazepine with hypnotic-sedative, anxiolytic, muscle relaxant, and anticonvulsant properties. It increases activity of GABA [28].

Fentanyl/fluanisone and midazolam are often the preferred drug combination of choice for surgical anesthesia of rodents and rabbits [23]. Anesthesia can be prolonged up to 8 hours in the rat by the administration of one-third of the original dose at 30–40 minute intervals [29–31]. The poor degree of muscle relaxation makes these drugs inappropriate for anything other than superficial surgery. The combination with a benzodiazepine (e.g., midazolam or diazepam) allows for dose reduction by 50–70%. This type of anesthesia can lead to tachycardia, hypotension, respiratory depression and polyuria [32].

Although frequently used in neurophysiological experiments [33], no mouse- or rat-model LUT studies using fentanyl-fluanisone-midazolam have been published to date. We performed cystometry under fentanyl-fluanisone-midazolam anesthesia and observed marked polyuria and continuous leakage of urine during bladder filling, suggestive of urethral sphincter relaxation (Unpublished data).

#### 3. Ketamine and xylazine

Ketamine is a noncompetitive NMDA receptor antagonist that provides analgesia and prevents central sensitization [34]. It causes a dissociative type of anesthesia with minimal cardiac and respiratory depression [15]. The absence of muscle relaxation makes ketamine a poor choice as the sole anesthetic for surgery [35].

Xylazine, a thiazole drug (N-(2,6-Dimethylphenyl)-5,6-dihydro-4H-1,3-thiazin-2-amine), is a strong α2-adrenergic agonist. Its mechanism of action is mediated by stimulation of the central α2-receptors; decreasing the release of norepinephrine and dopamine in the CNS and leading to sedation, muscle relaxation and decreased perception of painful stimuli. It is rapidly absorbed following intramuscular (i.m.) administration [36, 37]. Xylazine is used in a variety of species including the rat and mouse [38, 39] in conjunction with ketamine, to eliminate muscle rigidity [15, 17].

Cannon and Damaser [4] compared the effects of ketamine/xylazine (K/X) and urethane anesthetics on filling, voiding, and LPP in female rats. Rats were anesthetized with urethane (1.2 g/kg i.p.) or with K/X, 100 mg/kg and 15 mg/kg i.p. respectively. The data were compared to a control group, which underwent awake urodynamic studies. Both types of anesthesia reduced bladder capacity but showed no effect on voiding pressure and LPP. The results showed that both K/X and urethane exert effects on bladder function, but no effects on the urethral sphincter were observed.

K/X anesthesia does not eliminate the micturition reflex [40]. None of the mice anesthetized by the K/X combination developed urethral obstruction [41]. Studies have shown that other widely used combinations including ketamine/diazepam and ketamine/acepromazine do eliminate the micturition reflex [17].

#### 4. Propofol

Propofol is a substituted isopropylphenol, chemically distinct from the barbiturates, steroids, and imidazoles. As with other GABA agonists, propofol is a poor analgesic [42, 43]. The anesthetic properties of propofol are similar to those of the thiobarbiturates. Recovery from a single dose of propofol is rapid. Due to its minimal cumulative effect, it is often administered in a continuous intravenous infusion [44, 45]. Continuous intravenous infusion of propofol has been used in rats for combined cystometry and EUS EMG recording. No micturition reflex was noted after rats received 100%, 80%, or 60% of a previously reported anesthetic dose of propofol (1 mg/kg/min). At 40% of the standard propofol dose, a subset of rats showed reflex voiding, with bladder contractions and associated EUS EMG activity. The voiding efficiency was decreased when compared with that of rats anesthetized with urethane. It has been concluded that propofol anesthesia suppresses the micturition reflex in rats and therefore its use for rodent urodynamics is limited [46].

#### 5. Isoflurane

Isoflurane is an inhalation general anesthetic. It is used as the sole agent for induction and maintenance of general anesthesia [47]. The most likely sites of action in the CNS include inhibition of neurotransmitter-gated ion channels such as GABA, glycine and NMDA receptors [48, 49]. It has other sites of action within the spinal cord that induce skeletal muscle relaxation through the inhibition of NMDA-type glutamate and glycine receptors [47]. Isoflurane has limited effects on cardiac function, but it is a respiratory depressant and has vasodilatory effects [49]. The anesthesia induction and recovery are fast [50].

Chang and Havton compared urethane (1.2 g/kg s.c.) to isoflurane (2%–2.5%) and observed that micturition reflexes were differentially affected. Isoflurane was observed to cause prolonged bladder intercontractile intervals, reduced burst frequency, reduced firing frequency, decreased EUS EMG amplitude during voiding and filling. Other key functional aspects of bladder contractility were not found to be significantly different between the two experimental groups [51].

#### 6. Pentobarbital

Pentobarbital is a short-acting oxybarbiturate analog of barbituric acid. It has been used as a sedative–hypnotic, anesthetic, and anticonvulsant. The mechanism of action is similar to that of benzodiazepines and propofol in that GABA receptors are activated resulting in enhanced GABA binding and opening of transmembrane chloride channels, leading to cellular hyperpolarization within the CNS.

Xu et al. [52] evaluated the effects of pentobarbital on LUT function and defined an appropriate dose suitable for urodynamic studies in which recovery from anesthesia and long-term survival were needed. Rats in study groups received gradient doses of sodium pentobarbital i.p. Rats in the control group received urethane (1.2 g/kg i.p.). The EUS EMG was recorded simultaneously with cystometry and LPP measurement. Results revealed that micturition was normally induced in both the urethane and 32 mg/kg pentobarbital group. However, in those who received higher doses of pentobarbital, micturition failed to be induced. Instead, non-voiding contractions accompanied by EUS EMG tonic activity were observed. There were no significant differences in LPP or EUS EMG amplitude or frequency between the urethane and 32 mg/kg pentobarbital groups. This study confirmed significant dose-dependent effects of pentobarbital on LUT function and identified 32 mg/kg pentobarbital as the appropriate dose for the recovery of female rats after urodynamic testing, which enable the achievement of expected essential micturition undersatisfactory anesthesia.

A 40–50 mg/kg dose of pentobarbital is recommended for surgical anesthesia. Thus, 32 mg/kg will not be sufficient for studies which involve surgery. Moreover, pentobarbital has a prolonged recovery period, and is not recommended in rodents for recovery procedures [53]. Pentobarbital is no longer used for routine surgical anesthesia due to the narrow safety margin, low therapeutic index, habituation, and lack of an antidote [54, 55].

### II. Spinal anesthesia

Spinal anesthesia has been used in rats mostly to address the spinal mechanisms involved in the neuroregulation of the LUT. This method requires catheterization of the subarachnoid space. The catheter insertion could be performed through the atlanto-occipital membrane [56] or at the junction of the L5 and L6 lumbar vertebrae [57] under general inhalation [58] or injection anesthesia [59].

#### 1. Lidocaine

Lidocaine is a local anesthetic with an amide structure. Intrathecal (IT) lidocaine primarily blocks the generation and propagation of action potentials through direct binding of neuronal voltage-gated sodium channels, inhibiting excitation of nerve endings and producing analgesia by blocking conduction in peripheral nerves [60]. Mechanisms of action of lidocaine may also involve interactions with other ion channels, receptors (e.g., G protein-coupled receptors), and proteins that modify their activity (e.g. protein kinase A and C) [61].

Guerios et al. [62] performed a study in rats using intrathecal or intravesical administration of lidocaine (2%, 20 μl) prior to induction of chemical cystitis. Intrathecal lidocaine administered 15 minutes before intravesical injection of acrolein attenuated referred hyperalgesia associated with acrolein-induced cystitis. Wøien and colleagues [63] used IT injection of lidocaine to suppress the micturition reflex in a chronic rat model for testing new therapies for stress urinary incontinence. Lidocaine suppressed the micturition contractions allowing for LPP measurement and caused transient paraplegia in awake rats.

#### 2. Bupivacaine

Bupivacaine also has an amide structure, is four times stronger than lidocaine, and its duration of action is two to three times longer. It is also more lipophilic and penetrates further into myelinated motor fibers. IT bupivacaine was used to investigate changes in spinal mechanisms involved in detrusor hyperactivity. Repeated IT injection (50–100 μg) resulted in paralysis of the hind limbs and dribbling due to overflow incontinence (for 3–31 minutes), suggesting detrusor relaxation and no effects on EUS. IT administration of bupivacaine in rats with bladder outflow obstruction decreased micturition pressure and increased both bladder capacity and amplitude of spontaneous contractile activity [64].

#### 3. Fadolimidine

Fadolimidine is a α2-adrenoceptor agonist. Leino et al. studied effects of IT fadolmidine on kidney function, urodynamics and cardiovascular variables. In urodynamic studies, IT fadolimidine interrupted volume-evoked voiding cycles. At high concentrations it induced overflow incontinence. In addition, fadolimidine decreased heart rate and urine output in a dose-dependent manner and increased initial mean arterial pressure [65].

Summary of all above described anesthetic protocols and comparison of their differential effects on bladder capacity, filling and voiding pressure, LPP and EUS EMG activity are summarized in the Table 1.

**Table 1:** Summary of anesthetic protocols used for urodynamic studies of lower urinary tract in rodents.

Anesthetic	Animal	Dose & route of administration	Effects	Reference
**I. General anesthesia**				
Urethane	Rats (f)	1.2 g/kg, s.c.	○ Large-amplitude reflex bladder contractions of 30–40 cm H_2_O. ○ Smaller bladder capacity in CNS-intact anesthetized rats than in unanesthetized chronic spinal rats. ○ Reflex voiding, 42–74% of bladder volume.	[20]
	Mice (f)	1.2 g/kg s.c.	○ Delayed voiding pressure. ○ Lowered voided volume. ○ Higher contraction threshold pressure and maximum pressure. ○ Preservation of pulsatile high frequency oscillations activity with voiding.	[18]
	Rats (f/m)	3.2–4.0 mg/kg/min i.v. infusion	○ Preserved micturition reflex. ○ No reduction in micturition pressure.	[17]
	Rats (f)	0.6–1.2 g/kg i.v.	○ Decrease in maximal voiding pressure by 42%. ○ Decreased EUS EMG activity by 80%. ○ Increased micturition pressure threshold. ○ Increased postvoid residual volume. ○ Decreased voiding efficiency.	[22]
Ketamine/xylazine	Rats (f)	Ketamine	○ Decreased bladder capacity by 45%. ○ Unchanged voiding characteristics. ○ No significant difference in the LPP or threshold pressure. ○ Spontaneous non-voiding contraction occurred at a low bladder volume.	[4]
		100 mg/kg and		
		xylazine		
		15 mg/kg i.p.		
Propofol	Rats (f)	1 mg/kg/min i.v. continuous infusion	○ Marked suppression of bladder contractions. ○ Decreased voiding efficiency. ○ Increased functional bladder capacity. ○ Marked suppression of the EUS EMG activity.	[46]
Isoflurane	Rats (f)	2–2.5% i.h.	○ Marked suppression of bladder contractions. ○ Prolonged inter-contraction interval. ○ No significant differences in CMG parameters. ○ Reduced EUS EMG amplitude during the bladder filling. ○ Reduced frequency and amplitude of the EUS EMG activity during voiding.	[51]
Pentobarbital	Rats (f)	32 mg/kg,	*High doses (36 and 40 mg/kg)* ○ No micturition could be evoked. ○ Non-voiding bladder contractions. ○ Increased tonic activity on EUS-EMG during non-voiding bladder contractions. *Low dose (32 mg/kg)* ○ Preservation of the bladder function. ○ Prolonged micturition contraction. ○ Reduced frequency and duration of EUS-EMG bursting activity.	[52]
		36 mg/kg, or		
		40 mg/kg i.p.		
**II. Spinal anesthesia**				
Lidocaine	Rats (f)	2%, 20 μL i.t.	○ Suppression of micturition contractions. ○ Increased LPP.	[63]
Bupivacaine	Rats (f)	50–100 μg i.t.	○ Decrease in micturition pressure. ○ Increase in bladder capacity. ○ Dribbling incontinence due to urinary retention. ○ Increased amplitude of spontaneous contractions.	[64]
Fadolmidine	Rats (m)	1, 3, 10 and 30 μg/rat i.t.	○ Interrupted volume-evoked voiding cycles. ○ At analgesic doses, the above effects were mild. ○ Induced overflow incontinence at high concentrations.	[65]

Abbreviations: f, female; m, male; CNS, central nervous system; s.c., subcutaneous; i.p., intraperitoneal; i.t., intrathecal; i.v., intravenous; i.h., inhalation; K/X, ketamine/xylazine; LPP, leak point pressure; LUT, lower urinary tract; EUS, external urethral sphincter; EMG, electromyogram; CMG, cystometrograph.

## Discussion

When designing a urodynamic study under general anesthesia, the investigator should understand the effects of the anesthetic on all components of the urinary tract, most notably the smooth, striated muscle and innervation. It is often advantageous to repeat the urodynamic test in the same animal at baseline and after intervention to validate the disease model (e.g., bladder outflow obstruction or sphincter injury) or after administration of the experimental treatment. If repeat studies are planned in the same animal, the anesthetic must be free of long-term side effects.

Anesthetics affect the function of the LUT to a different degree. For decades, urethane has been the preferred drug of choice, due to its mild effects on LUT function. Several studies compared different anesthetics to urethane and did confirm this consensus. However, when urodynamic findings obtained under urethane and K/X were compared to awake cystometry, the only parameter which was affected by both types of anesthesia was functional bladder capacity. No difference was seen in the effects of the two anesthetics on the bladder pressures and urethral sphincter function. We observed that 60% of the K/X dose recommended for surgical anesthesia is sufficient when performing LPP measurements. This is significant because, urethane which to date has been extensively used in LPP studies has carcinogenic and mutagenic effects and its use should be avoided when possible. In addition, due to its toxicity to animals it must be reserved for non-survival experiments [53].

Isoflurane is a frequently used, volatile anesthetic that provides reliable surgical anesthesia and fast recovery. It has been used in rodent urodynamic studies despite its effects on both bladder and urethral function. These effects could be minimized by using a reduced dose. We have conducted LPP measurements under both isoflurane or K/X and observed that even at low concentrations (1.5% with oxygen as carrier), isoflurane lowers the LPP more than K/X. Isoflurane could be used in situations when the testing period is short and a fast recovery from anesthesia is advantageous.

Similarly, it has been documented that there are possible interactions between anesthetic agents and detrusor contractile activity. Ceran and colleagues evaluated the effects of 3 different intravenous anesthetics: propofol, ketamine, and midazolam on detrusor contractile responses *in vitro*. Results demonstrated that depressant effects of midazolam on the contractile activity were found to be more significant than ketamine and propofol [66].

## Conclusion

Bladder filling and micturition depend on the integrated function of the bladder, urethra and pelvic floor as well as a number of tissue types including smooth and striated muscle, urothelium and nervous system. Different anesthetics affect these components to varying degrees. The goal of this communication was to summarize existing literature and discuss advantages and disadvantages of individual or combination anesthetic protocols to help standardize future experiments. Various types of anesthesia used by different research groups to perform urodynamic studies allowed us to summarize the effects of individual anesthetics on different components of the LUT. The least unwanted effects were observed with ketamine and xylazine. Other anesthetics however can be used in select experiments.

## Supporting information

S1 Table The Preferred Reporting Items for Systematic Reviews and Meta-Analyses (PRISMA) guidelines were followed on data identification, screening, eligibility, and inclusion of studies. The PRISMA checklist for this review is provided in a (S1 Table).
(DOCX)
